# Grossesses extra utérines successives et bilharziose tubaire chez une touriste française

**DOI:** 10.11604/pamj.2016.23.18.8845

**Published:** 2016-01-26

**Authors:** Justine Laroche, Nicolas Mottet, Marianna Malincenco, Catherine Gay, Pierre Yves Royer, Didier Riethmuller

**Affiliations:** 1 Service de Gynécologie, Hôpital Nord Franche Comté, Belfort, France; 2 Service de Gynécologie, Hôpital Régional Universitaire de Besançon, France; 3 Service de Maladies Infectieuses, Hôpital Nord Franche Comté, Belfort, France

**Keywords:** Grossesse extra utérine, bilharziose tubaire, trompe, Ectopic pregnancy, tubal schistosomiasis, tube

## Abstract

La bilharziose est la seconde endémie parasitaire mondiale et ses atteintes génito urinaires sont bien décrites. Les grossesses ectopiques (GEU) sur obstruction tubaire par les œufs de bilharziose sont rapportées dans les populations africaines. La bilharziose affecte aussi les voyageurs mais l'atteinte de l'appareil génital féminin est plus rare. Nous rapportons un cas exceptionnel de deux GEU successives sur bilharziose tubaire chez une patiente d'origine Française, sept ans après un voyage touristique au Mali, la première découverte sur pièce de salpingectomie et la seconde ayant nécessité une salpingotomie controlatérale avec une injection de méthotrexate, deux mois plus tard.

## Abstract (en)

Schistosomiasis is the second endemic parasitic disease in the world and is a common cause of urogenital infections. Ectopic pregnancies due to tubal obstruction by schistosoma's eggs are usually reported in Africa. Schistosomiasis also affects travelers but infection of the female genital tract is less frequently described. We report an unusual clinical case of two successive ectopic pregnancies with tubal schistosomiasis in a French woman, seven years after a travel to Mali. The first event was discovered after histologic examination of salpingectomy and the second event required a controlateral salpingotomy with an injection of methotrexate, two months later.

## Introduction

La bilharziose ou schistosomiase est une maladie parasitaire à vers plat d'Afrique et d'Asie à transmission urinaire ou fécale dans les eaux douces. On compte 230 millions de personnes atteintes au travers du monde. Il existe trois types de vers: schistosoma Mansoni (tropisme intestinal), Haematobium (tropisme urinaire) et Japonicum (tropisme artério veineux). Dans les eaux douces, la larve de schistosoma pénètre par voie transcutanée, migre dans la circulation sanguine et y devient adulte. Elle pond des œufs qui migrent à travers la paroi d'un organe creux (intestin, vessie) et sont éliminés dans les selles ou les urines. Le traitement est médical et repose sur une prise unique de Praziquantel. La contamination génitale se fait par le biais d'anastomoses vasculaires entre la vessie et l'appareil génital féminin. Les œufs de schistosoma peuvent se nicher dans les trompes et entrainent une fibrose classique avec une inflammation provoquant l'obstruction de celles-ci. Les grossesses extra utérines (GEU) d'origine parasitaire sont fréquentes en Afrique et sont souvent découvertes devant un tableau d’ hémopéritoine. La bilharziose atteint aussi les voyageurs avec une atteinte urinaire plus fréquente (hématurie, infections urinaires) que l'atteinte génitale. Nous rapportons un cas exceptionnel de deux GEU successives sur bilharziose tubaire chez une patiente d'origine Française, sept ans après un voyage touristique au Mali. La première a été découverte sur pièce de salpingectomie, la seconde GEU, au cycle suivant, a bénéficié d'une salpingotomie controlatérale avec une injection de méthotrexate complémentaire.

## Patient et observation

Il s'agit d'une patiente de 26 ans, G3P1, d'origine Française, résidant en Franche Comté dans le Nord Est de la France. On note dans ses antécédents: une grossesse spontanée avec un accouchement voie basse en 2013, une fausse couche en 2014. Elle a effectué deux voyages touristiques: au Mali en 2008 puis en Guyane en 2010.

Elle consulte les urgences gynécologiques de l'hôpital Nord Franche Comté le 9 juin 2015 pour douleurs en fosse iliaque gauche avec un épisode de lipothymie, retard de règles et le dosage de βHCG fait en ville la veille est à 1700 UI/L. A l’échographie, on retrouve une vacuité utérine et une masse latéro utérine gauche de 40*33mm avec un épanchement du cul de sac de Douglas avec un dosage des βHCG à 1797UI/L. On suspecte une GEU gauche: on décide d'une prise en charge chirurgicale le jour même par salpingectomie gauche par coelioscopie pour une GEU tubaire gauche non rompue. L'analyse anatomopathologique de la pièce opératoire retrouvait des granulomes bilharziens autour d'oeufs de shistosomoses centrant des nodules de sclérose. Les œufs sont en involution et calcifiés. Les suites opératoires sont simples et la patiente sort d'hospitalisation avec une contraception et un suivi en maladies infectieuses avec un bilan négatif (absence d’œufs dans les urines et absence d'hyperéosinophilie). Le traitement médical par praziquantel est prescrit.

Le 30 juillet 2015, Un dosage des βHCG est réalisé avant la prise de praziquantel devant l'aménorrhée depuis l'intervention chirurgicale: ils sont à 973UI/L. La patiente consulte aux urgences gynécologiques deux jours plus tard pour douleurs abdominales. A l’échographie, on retrouve une vacuité utérine et une masse latéro utérine droite avec un épanchement du cul de sac de Douglas. Le dosage des βHCG est à 1295UI/L. Devant la suspicion de GEU controlatérale, le traitement est chirurgical par une salpingotomie droite par coelioscopie pour une GEU tubaire droite non rompue. L'histologie du produit d'aspiration montre du trophoblaste. Les suites opératoires sont simples et la patiente sort d'hospitalisation avec une décroissance des BHCG à réaliser jusqu’à négativation. Elle reçoit son traitement par praziquantel. A J 12 post opératoire, le taux de βHCG ré-ascensionne à 916 UI/L (βHCG à 715UI/L au dernier bilan).

Un contrôle échographique retrouve une petite image latéro ovarienne droite de 18*11mm, le dosage de contrôle des βHCG est à 1027UI/L 48h après. On décide d'un traitement médical par une injection de Méthrotrexate 75mg par voie intra musculaire. La cinétique de la décroissance est bonne et permet sa négativation. Les échographies de contrôle sont sans particularité.

## Discussion

Les atteintes génitales de la bilharziose sont connues et peuvent toucher les ovaires, l'utérus, la vulve, le vagin, le col utérin et les trompes. Les grossesses extra utérines (le plus souvent rompues avec un hémopéritoine) sont fréquemment rapportées dans la littérature mais uniquement dans les populations africaines [1, 2]. Néanmoins un cas en Inde et un au Brésil ont été décrits [3]. Un seul cas de GEU bilatérale synchrone est relaté chez une femme Nigérienne [2]. Peu d'articles relatent les atteintes génitales chez les voyageurs femmes, elles sont majoritairement urinaires [4]. On retrouve quelques cas de bilharziose tubaire: l'une chez une britannique et l'autre chez une française, découverts sur pièce opératoire de salpingectomie pour douleurs pelviennes [5, 6]. Deux cas de bilharziose vulvaire ont été décrits dont l'une chez une britannique également après s’être baignée au lac Malawi [7, 8]. Il n'est rapporté que deux cas de GEU sur bilharziose tubaire chez un voyageur: il s'agit d'une Australienne de 34 ans, qui avait voyagé une dizaine d'années plus tôt au Lac Victoria et Malawi et une Anglaise de 37 ans suspecté en per coelioscopie pour suspicion de GEU [9, 10].

Concernant notre patiente, la taille échographique de la masse latéro utérine et de l’épanchement nous a emmené à choisir le traitement chirurgical en première intention et ainsi à découvrir fortuitement le diagnostic de bilharziose tubaire chez une patiente totalement asymptomatique jusque là. Puis elle récidive dès le cycle suivant sur la trompe controlatérale devant l'absence de reprise de contraception. Il n’ a pas été décrit de cas de GEU successives à l'heure actuelle sur bilharziose. Devant le pronostic de fertilité et l'absence de littérature, on décide de tenter un traitement conservateur par salpingotomie, et de compléter le geste par une injection de méthotrexate devant une réascension des βHCG. Il n'existe aucune publication utilisant un traitement conservateur et/ou médical par méthotrexate pour une GEU sur bilharziose tubaire. La prochaine grossesse de cette patiente sera sous étroite surveillance et la récidive d'une GEU imposera une salpingectomie droite, et la nécessité du recours à la procréation médicalement assistée.

Cependant, il est intéressant de constater que la patiente a mené une grossesse spontanée sans difficulté, des années après son retour du Mali. Deux hypothèses en découlent de ce fait: il existe une certaine perméabilité tubaire résiduelle ou la fibrose des tissus générée par la présence des œufs s'aggrave avec le temps entrainant une obstruction définitive tardive.

## Conclusion

La GEU sur bilharziose tubaire est une pathologie du voyageur exceptionnelle en France. L'atteinte peut être bilatérale sans être synchrone. Son diagnostic est fait à postiori sur pièce de salpingectomie. Un traitement complémentaire par praziquantel est nécessaire, en prise unique. Le recours à un traitement conservateur associé à du methotrexate s'est avéré efficace, dans le seul but de préserver la fertilité, mais sans pour autant, avoir de recul sur la th érapeutique dans ce cas de figure.
